# Comparative genomic reconstruction of transcriptional networks controlling central metabolism in the *Shewanella* genus

**DOI:** 10.1186/1471-2164-12-S1-S3

**Published:** 2011-06-15

**Authors:** Dmitry A Rodionov, Pavel S Novichkov, Elena D Stavrovskaya, Irina A Rodionova, Xiaoqing Li, Marat D Kazanov, Dmitry A Ravcheev, Anna V Gerasimova, Alexey E Kazakov, Galina Yu Kovaleva, Elizabeth A Permina, Olga N Laikova, Ross Overbeek, Margaret F Romine, James K Fredrickson, Adam P Arkin, Inna Dubchak, Andrei L Osterman, Mikhail S Gelfand

**Affiliations:** 1 Sanford-Burnham Medical Research Institute, La Jolla, California, USA; 2 Institute for Information Transmission Problems, Russian Academy of Sciences, Moscow, Russia; 3 Lawrence Berkeley National Laboratory, Berkeley, California, USA; 4 Faculty of Bioengineering and Bioinformatics, Moscow State University, Moscow, Russia; 5 State Scientific Center GosNIIGenetika, Moscow, Russia; 6 Fellowship for Interpretation of Genomes, Burr Ridge, Illinois, USA; 7 Biological Sciences Division, Pacific Northwest National Laboratory, Richland, Washington, USA; 8 Department of Energy Joint Genome Institute, Walnut Creek, California, USA

## Abstract

**Background:**

Genome-scale prediction of gene regulation and reconstruction of transcriptional regulatory networks in bacteria is one of the critical tasks of modern genomics. The *Shewanella* genus is comprised of metabolically versatile gamma-proteobacteria, whose lifestyles and natural environments are substantially different from *Escherichia coli* and other model bacterial species. The comparative genomics approaches and computational identification of regulatory sites are useful for the *in silico* reconstruction of transcriptional regulatory networks in bacteria.

**Results:**

To explore conservation and variations in the *Shewanella* transcriptional networks we analyzed the repertoire of transcription factors and performed genomics-based reconstruction and comparative analysis of regulons in 16 *Shewanella* genomes. The inferred regulatory network includes 82 transcription factors and their DNA binding sites, 8 riboswitches and 6 translational attenuators. Forty five regulons were newly inferred from the genome context analysis, whereas others were propagated from previously characterized regulons in the Enterobacteria and *Pseudomonas* spp.. Multiple variations in regulatory strategies between the *Shewanella* spp. and *E. coli* include regulon contraction and expansion (as in the case of PdhR, HexR, FadR), numerous cases of recruiting non-orthologous regulators to control equivalent pathways (e.g. PsrA for fatty acid degradation) and, conversely, orthologous regulators to control distinct pathways (e.g. TyrR, ArgR, Crp).

**Conclusions:**

We tentatively defined the first reference collection of ~100 transcriptional regulons in 16 *Shewanella* genomes. The resulting regulatory network contains ~600 regulated genes per genome that are mostly involved in metabolism of carbohydrates, amino acids, fatty acids, vitamins, metals, and stress responses. Several reconstructed regulons including NagR for N-acetylglucosamine catabolism were experimentally validated in *S. oneidensis* MR-1. Analysis of correlations in gene expression patterns helps to interpret the reconstructed regulatory network. The inferred regulatory interactions will provide an additional regulatory constrains for an integrated model of metabolism and regulation in *S. oneidensis* MR-1.

## Background

Fine-tuned regulation of gene expression in response to extracellular and intracellular signals is a key mechanism for successful adaptation of microorganisms to changing environmental conditions. Activation and repression of gene expression in bacteria is usually mediated by DNA-binding transcription factors (TFs) that specifically recognize TF-binding sites (TFBSs) in upstream regions of target genes, and also by various regulatory RNA structures including *cis*-acting metabolite-sensing riboswitches and attenuators encoded in the leader regions of target genes. Genes and operons directly co-regulated by the same TF or by an RNA structure are considered to belong to a *regulon*. All regulons taken together form the transcriptional regulatory network (TRN) of the cell. TFs form more than 50 different protein families and constitute around 5-10% of all genes in an average bacterial genome, and their respective regulons cover a substantial fraction of bacterial TRNs [1].

Traditional experimental methods for the analysis of transcriptional gene regulation and characterization of TFBSs provided a foundation for the current understanding of regulatory interactions [2]. However, taken alone, they are limited in productivity (the scale) and feasibility (often restricted to a few model organisms). High-throughput transcriptome approaches opens new opportunities for measuring the expression of thousands of genes in a single experiment [3]. The microarray technology has been successfully used to explore transcriptional responses in several bacteria. However, convoluted regulatory cascades, multi-TF regulation of certain genes, and various indirect effects on the transcription and abundance of mRNA make the observed regulatory responses too complex for a direct top-down analysis. The chromatic immunoprecipitation approach is now increasingly used for the investigation of genome-wide DNA-binding of global TFs in bacteria [3]. At the same time, a growing number of complete prokaryotic genomes allows us to extensively use comparative genomics approaches to infer conserved *cis*-acting regulatory elements (e.g. TFBSs and riboswitches) in regulatory networks of numerous groups of bacteria ([4-15], also reviewed in [1]). These and other previous studies enabled us to define and prototype a general workflow of the “knowledge-driven” approach for the comparative-genomic reconstruction of regulons. Two major components of this analysis are (i) propagation of previously known regulons from model organisms to others and (ii) *ab initio* prediction of novel regulons (see Methods for more details). This approach is different, and in many ways complementary to the two most common alternative approaches to the TRN reconstruction: (i) the “data-driven” approach, top-down regulatory network reconstruction from microarray data [16]; and (ii) the “computation-driven” approach, *ab initio* automated identification and clustering of conserved DNA motifs [17] .

**Figure 1:**
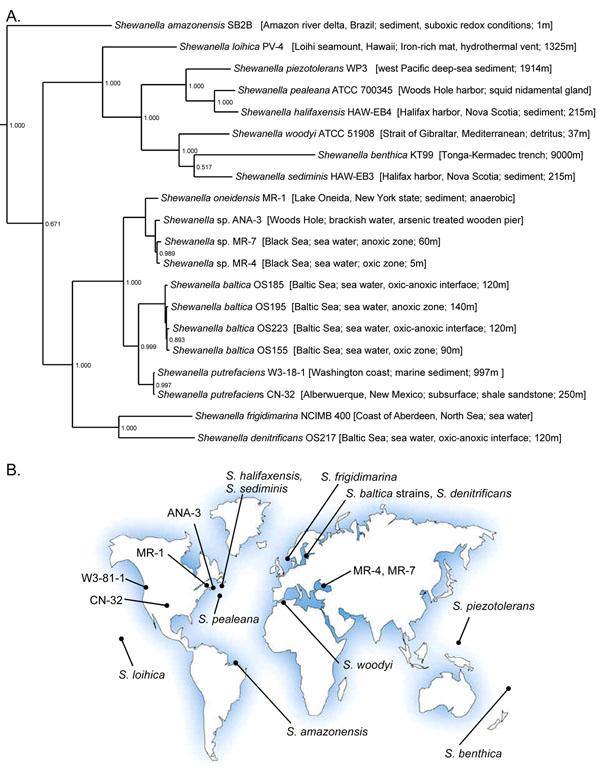
Phylogenetic tree, isolation site characteristics and the geographic origin of 20 *Shewanella* species with available genomes. The tree was constructed using the concatenated alignment of ~78 universal prokaryotic proteins in the MicrobesOnline database http://www.microbesonline.org/cgi-bin/speciesTree.cgi.

*Shewanella* spp. are Gram-negative facultative anaerobic γ-proteobacteria characterized by a remarkable versatility in using a variety of terminal electron acceptors for anaerobic respiration (reviewed in [18]). Isolated from various aquatic and sedimentary environments worldwide, the *Shewanella* demonstrate diverse metabolic capabilities and adaptation for survival in extreme conditions (Fig. 1) [19]. Although the model species *Shewanella oneidensis* MR-1 is a subject of extensive genetics and physiological studies, as well as genome-scale transcriptomics and proteomics approaches [18,20-22], our experimental knowledge of transcriptional regulation in *S. oneidensis* is limited to the Fur, ArcA, TorR, Crp, and EtrA (Fnr) TFs controlling iron metabolism and anaerobic respiration [23-29]. In addition, the novel NrtR regulon for NAD cofactor metabolism was inferred by comparative genomics and experimentally validated in *S. oneidensis*[11].

Availability of multiple closely-related genomes from the *Shewanella* genus (Fig. 1) provided a basis for the reconstruction of the metabolic and regulatory networks using comparative genomics. Recently, we have applied the comparative genomic approach to predict novel pathways and regulons for the *N-*acetylglucosamine and lactate utilization [30,31], and to reconstruct two novel regulons for the fatty acid and branched-chain amino acid utilization pathways in *Shewanella* spp. [4]. In this study, we have extended our previous analysis towards the detailed reconstruction of ~100 transcriptional regulons in 16 *Shewanella* species with completely sequenced genomes. The identified TRN contains over 450 regulated genes per genome, mostly covering the central and secondary metabolism and stress response pathways. The comparative analysis of the reconstructed regulons revealed many aspects of the metabolic regulation in the *Shewanella* that are substantially different from the established TRN model of *Escherichia coli*.

## Results

### Repertoire of transcription factors in the *Shewanella* spp

**Figure 2:**
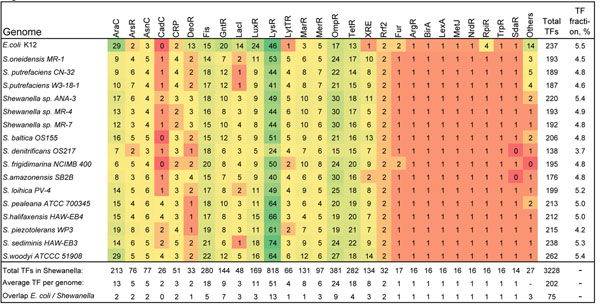
Distribution by protein families of predicted DNA-binding transcription factors in the *Shewanella* genomes.

Previous comparative analysis revealed extensive gene content diversity among 10 *Shewanella* genomes [32]. To gain further insight into the scale of the TRN diversity in this lineage, we analyzed the repertoire of DNA-binding TFs encoded in 16 complete *Shewanella* genomes (Additional file 1). The total number of TFs in individual species varies broadly, from 138 TFs in *S. denitrificans* to 262 TFs in *S. woodyi*, with an average of ~200 TFs per genome (Fig. 2). 95% of all TFs of the *Shewanella* belong to 17 major protein families with at least two distinct members per genome. At that, the total number of TFs in most of these families varies significantly among the *Shewanella* spp. The largest TF families are LysR, OmpR, Fis, TetR, AraC, and LuxR (>10 TFs per genome on average). Among the remaining 14 families of TFs, mostly represented by single members in the genomes (without paralogs), the Fur, ArgR, BirA, LexA, MetJ, NrdR, RpiR, and TrpR families are universally conserved in the *Shewanella* (Fig. 2). A significant reduction of the TF repertoire is a unique feature of *S. denitrificans*, which has limited anaerobic growth capabilities due to massive gene loss in course of ecological specialization [32].

The 3,228 predicted TFs in 16 *Shewanella* genomes were clustered into 686 orthologous groups (Additional file 1), among which only 63 TFs (9%) were universally conserved in all genomes (the core TF set), 320 TFs (47%) were found in at least two genomes (variable TFs), whereas the remaining TFs (303 or 44%) were strain-specific (Additional file 1). Although the genomes of the *Shewanella* spp. and *E. coli* demonstrate a similar repertoire and size of TF protein families, only 73 (30%) TFs from *E. coli* have orthologs in at least one *Shewanella* genome (Fig. 2). The group of 34 TFs that are present in the *Shewanella* core TF set (Additional file 2) and conserved between *E. coli* and the *Shewanella* spp. (Additional file 3) is enriched by regulators controlling the metabolism of amino acids (ArgR, AsnC, CysB, GcvA, IlvY, MetJ, MetR, TrpR, TyrR), fatty acids (FabR, FadR), cofactors (BirA, IscR), deoxynucleosides (NrdR), nitrogen (NtrC), phosphate (PhoB), iron (Fur), central carbohydrate metabolism (HexR, PdhR), stress responses (CpxR, LexA, NhaR, NsrR), and global regulators (ArcA, Crp, Fis, Fnr, and Lrp). The group of strain-specific *Shewanella* regulators with orthologs in *E. coli* contains 5 known regulators for local carbohydrate utilization pathways (AlgR, NanR, DgoR, GalR, GntR) that were possibly acquired together with the target metabolic pathway genes via lateral gene transfer events [33]. Near 1/2 of strain-specific TFs of the *Shewanella* spp. belong to two protein families, LysR and AraC (96 and 50 TFs, respectively), that were likely expanded via gene duplication in course of ecological adaptation of individual species.

### Comparative analysis of transcriptional regulation in the *Shewanella* spp

To infer TRNs in the *Shewanella* spp., we used the integrative comparative genomics approach that combines identification of TFs and candidate TFBSs with cross-genomic comparison of regulons and with the genomic and functional context analysis of candidate target genes. We analyzed 16 *Shewanella* genomes and inferred regulons for 82 orthologous groups of TFs that split into two groups: 41 regulators with experimentally characterized orthologs in *S. oneidensis* or other γ-proteobacteria (Table 1), and 41 novel regulators without characterized orthologs in any species (Table 2). The genomic and functional content of the reconstructed TF regulons from both groups, as well as of the regulons controlled by known RNA regulatory elements (8 riboswitches and 6 transcriptional attenuators), is summarized in Additional file 4 and briefly described below. These data, in conjunction with the detailed information about DNA binding motifs and individual TFBSs, were compiled into the *Shewanella* collection of regulons that was uploaded to the RegPrecise database http://regprecise.lbl.gov[34].

**Table 1:** Previously known TF regulons reconstructed in *Shewanella* spp.

TF name	*S.oneidensis* MR-1	*S. putrefaciens* CN-32	*S.putrefaciens* W3-18-1	*Shewanella* sp. ANA-3	*Shewanella* sp. MR-4	*Shewanella* sp. MR-7	*S. baltica* OS155	*S. denitrificans* OS217	*S. frigidimarina* NCIMB 400	*S.amazonensis* SB2B	*S. loihica* PV-4	*S. pealeana* ATCC 700345	*S.halifaxensis* HAW-EB4	*S. piezotolerans* WP3	*S. sediminis* HAW-EB3	*S.woodyi* ATCCC 51908	*Pseudomonas spp.*	*E.coli*	Regulon functional role
AgaR	-	-	-	+	+	+	-	-	-	+	-	-	-	-	-	-	-	rs	GalNAc utilization
ArgR	+	+	+	+	+	+	+	+	+	+	+	+	+	+	+	+	-	rs	Arginine biosynthesis
BetI	-	-	-	-	-	-	+	+	+	-	-	+	+	+	+	+	+	rs	Osmotic protection
BirA	+	+	+	+	+	+	+	+	+	+	+	+	+	+	+	+	+	rs	Biotin biosynthesis
Crp	r	+	+	+	+	+	+	+	+	+	+	+	+	+	+	+	+	rs	Global regulon
CueR	+	+	+	+	+	+	+	+	-	+	-	+	+	+	+	+	rs	rs	Copper efflux
Dnr	-	-	-	-	-	-	-	+	-	+	+	-	-	-	-	-	rs	-	Denitrification
FabR	+	+	+	+	+	+	+	+	+	+	+	+	+	+	+	+	+	rs	Fatty acid biosynthesis
FadR	+	+	+	+	+	+	+	+	+	+	+	+	+	+	+	+	-	rs	Fatty acid degradation
Fnr	r	+	+	+	+	+	+	+	+	+	+	+	+	+	+	+	+	rs	Global regulon
Fur	rs	+	+	+	+	+	+	+	+	+	+	+	+	+	+	+	rs	rs	Iron homeostasis
GalR	-	-	-	-	-	-	-	-	-	-	-	-	-	-	-	+	-	rs	Galactose utilization
GcvA	+	+	+	+	+	+	+	+	+	+	+	+	+	+	+	+	-	rs	Glycine metabolism
GlmR	+	+	+	+	+	+	+	+	+	+	+	+	+	+	+	+	r	-	LPS synthesis
GntR	-	-	-	-	-	-	+	-	-	-	-	-	-	-	-	-	+	rs	Gluconate utilization
HexR	+	+	+	+	+	+	+	+	+	+	+	+	+	+	+	+	rs	+	Central sugar metabolism
HutC	+	+	+	+	+	+	+	+	+	+	+	+	+	+	+	+	r	-	Histidine utilization
IlvY	+	+	+	+	+	+	+	+	+	+	+	+	+	+	+	+	-	rs	Isoleucine-valine synthesis
IscR	+	+	+	+	+	+	+	+	+	+	+	+	+	+	+	+	+	rs	Fe-S cluster assembly
LexA	+	+	+	+	+	+	+	+	+	+	+	+	+	+	+	+	rs	rs	DNA damage stress
MetJ	+	+	+	+	+	+	+	+	+	+	+	+	+	+	+	+	-	rs	Methionine biosynthesis
MetR	+	+	+	+	+	+	+	+	+	+	+	+	+	+	+	+	+	rs	Methionine biosynthesis
ModE	+	-	-	+	+	+	+	-	-	-	-	-	-	-	-	-	+	rs	Molybdenium metabolism
NanR	-	-	-	-	-	-	-	-	-	-	-	+	-	-	-	-	-	rs	Sialic acid utilization
NarP	+	+	+	+	+	+	+	-	+	+	+	+	+	+	+	+	-	rs	Nitrate/nitrite respiration
NhaR	+	+	+	+	+	+	+	+	+	+	+	+	+	+	+	+	-	rs	Osmotic stress protection
NikR	-	-	-	-	-	-	-	-	-	-	-	+	+	-	+	-	+	rs	Nickel uptake
NorR	-	+	+	+	+	+	-	-	+	+	+	+	+	+	+	+	+	rs	Nitrosative stress
NrdR	+	+	+	+	+	+	+	+	+	+	+	+	+	+	+	+	+	rs	Nucleotide metabolism
NrtR	rs	+	+	-	-	-	-	-	-	-	-	-	-	-	-	-	+	-	Nicotinamide utilization
NsrR	+	+	+	+	+	+	+	+	+	+	+	+	+	+	+	+	-	rs	Nitrosative stress
NtrC	+	+	+	+	+	+	+	+	+	+	+	+	+	+	+	+	rs	rs	Nitrogen assimilation
PdhR	+	+	+	+	+	+	+	+	+	+	+	+	+	+	+	+	-	rs	Pyruvate metabolism
PsrA	+	+	+	+	+	+	+	+	+	+	+	+	+	+	+	+	rs	-	Fatty acid degradation
RbsR	-	-	-	-	-	-	-	-	-	-	-	+	+	-	-	-	+	rs	Ribose utilization
SdaR	+	+	+	+	+	+	+	-	+	-	+	+	+	+	+	+	+	r	Glycerate utilization
SoxR	-	-	-	-	-	-	-	+	+	+	+	-	-	-	-	+	+	rs	Superoxide stress
TorR	rs	-	-	+	+	+	+	-	+	+	+	+	+	+	+	+	-	rs	TMAO respiration
TrpR	+	+	+	+	+	+	+	+	+	+	+	+	+	+	+	+	-	rs	Amino acid metabolism
TyrR	+	+	+	+	+	+	+	+	+	+	+	+	+	+	+	+	rs*	rs	Amino acid metabolism
ZntR	+	+	+	+	+	+	+	+	+	+	+	+	+	+	+	+	-	rs	Zinc efflux

The presence of orthologous transcription factors is shown by '+', 'r' and 'rs', whereas its absence is denoted by '-'. TFs with previously characterized target genes in model species are denoted by 'r'. TFs with previously known TFBSs at their target genes are denoted by 'rs'. Ortholog of TyrR in *Pseudomonas* spp. that was characterized as the phenylalanine and tyrosine catabolism regulator PhhR is marked by asterisk.

**Table 2:** Novel TF regulons predicted and reconstructed in *Shewanella* spp.

TF name	*S.oneidensis* MR-1	*S. putrefaciens* CN-32	*S.putrefaciens* W3-18-1	*Shewanella* sp. ANA-3	*Shewanella* sp. MR-4	*Shewanella* sp. MR-7	*S. baltica* OS155	*S. denitrificans* OS217	*S. frigidimarina* NCIMB 400	*S.amazonensis* SB2B	*S. loihica* PV-4	*S. pealeana* ATCC 700345	*S.halifaxensis* HAW-EB4	*S. piezotolerans* WP3	*S. sediminis* HAW-EB3	*S.woodyi* ATCCC 51908	Regulon functional role
*A. Regulons inferred from the analysis of metabolic pathways*																	
AlgR*	-	-	-	-	-	-	-	-	+	-	-	-	-	-	-	-	Hexuronate utilization
AraR*	-	+	+	+	+	+	-	-	-	-	-	-	-	-	-	-	Arabinose utilization
BglR*	-	-	-	-	-	-	+	+	+	+	-	-	-	+	-	+	Beta-glucoside utilization
HmgR*	+	+	+	+	+	+	+	+	+	+	+	+	+	+	+	+	Tyrosine degradation
HypR*	+	+	+	+	+	+	+	+	+	+	+	+	+	+	+	+	Hydroxyproline utilization
LiuR	+	+	+	+	+	+	+	+	+	+	+	+	+	+	+	+	Amino acid utilization
LldR*	+	+	+	+	+	+	+	-	+	+	+	+	+	+	+	-	Lactate utilization
MalR*	+	-	-	+	+	+	+	+	+	+	+	-	-	+	-	+	Maltodextrin utilization
ManR1*	-	-	-	-	-	+	-	-	-	+	-	-	-	-	-	-	Mannose utilization
ManR2*	-	-	-	-	-	-	-	-	-	+	-	-	-	-	-	-	Mannose utilization
MtlR2	-	-	-	-	-	-	-	-	+	-	-	-	-	-	-	-	Mannitol utilization
NagR	+	+	+	+	+	+	+	+	-	+	+	+	+	+	+	+	GlcNAc utilization
PflR*	-	+	+	-	-	-	-	-	+	+	+	+	+	+	+	+	Formate metabolism
PrpR*	+	+	+	+	+	+	+	+	+	+	+	+	+	+	+	+	Propionate utilization
PUR*	+	+	+	+	+	+	+	+	+	+	+	+	+	+	+	+	Purine biosynthesis
ScrR*	-	-	-	+	+	+	+	-	+	-	-	-	-	-	-	-	Sucrose utilization
TreR*	-	-	-	-	-	-	-	-	+	-	-	-	-	-	-	+	Trehalose utilization
XltR*	-	-	-	-	-	-	-	-	-	-	-	+	+	-	-	-	Xylitol utilization
*B. Regulons inferred from the analysis of chromosomal gene clusters*																	
AzrR*	+	+	+	+	+	+	+	-	-	+	+	+	+	+	+	+	Superoxide stress
CalR*	+	+	+	+	+	+	+	+	+	+	+	+	+	+	+	+	Aromatics utilization
CueR2	-	+	-	-	-	-	-	-	+	-	+	-	-	-	-	-	Copper efflux
DeoR*	+	+	+	+	-	-	-	-	-	-	-	+	+	+	+	+	Nucleoside utilization
PnuR*	+	+	+	-	-	-	+	-	-	-	-	-	-	-	-	-	NAD metabolism
SO0072	+	+	+	+	+	+	+	+	+	+	+	+	-	+	-	+	ABC efflux transporter
SO0082	+	-	-	-	+	-	+	-	+	-	-	-	-	-	-	-	Benzoate degradation
SO0193	+	+	+	+	+	+	+	-	+	+	+	-	-	-	-	-	Phospholipid synthesis
SO0734	+	+	+	-	-	-	+	-	-	-	-	-	-	-	-	-	hypothetical transporter
SO1393	+	-	-	+	+	+	-	-	-	-	+	-	-	-	-	-	hypothetical
SO1415	+	-	-	-	+	-	-	-	-	-	+	+	+	-	+	-	flavocytochrome c
SO1578	+	-	-	+	+	+	+	-	-	+	+	-	-	-	-	+	Glutathione detoxification
SO1703	+	+	+	+	+	+	+	+	-	+	+	+	+	+	+	-	multidrug efflux
SO1758	+	+	+	+	+	+	+	-	-	+	+	-	-	-	+	+	hypothetical
SO2282	+	+	+	+	+	+	+	-	-	+	-	+	+	+	-	+	Amino acid efflux
SO3277	+	+	+	+	+	+	+	+	+	+	+	+	+	+	+	+	multidrug efflux
SO3385	+	+	+	+	+	+	+	-	-	-	-	-	-	-	-	-	hypothetical
SO3393	+	+	+	+	+	+	+	+	+	+	+	-	-	+	+	+	xenobiotic reductase
SO3494	+	+	+	+	+	+	+	+	+	-	-	-	-	-	-	-	multidrug efflux
SO3627	+	-	-	-	-	-	-	-	-	-	-	-	-	-	+	-	flavocytochrome c
SO4326	+	-	-	+	-	-	-	-	-	+	-	+	+	+	+	+	multidrug efflux
SO4468	+	+	+	+	+	+	+	+	-	+	-	-	-	+	+	-	hypothetical
SO4705	+	+	+	+	+	+	+	+	+	+	+	+	+	+	+	+	hypothetical

The new TF names introduced in this work are marked by asterisks.

#### Reconstruction of regulons for previously characterized regulators

Our general strategy of reconstructing regulons controlled by known TFs in a novel taxonomic group consists of the following steps: (i) search for orthologous TFs, (ii) collecting known target genes and TFBSs in a model genome, (iii) identifying orthologous target genes in the analyzed genomes and extracting their upstream regions, iv) application of a pattern recognition program, then constructing positional weight matrices (PWMs) and comparison of the newly identified TFBS motifs with the previously known sites/motifs in a model genome, v) search for additional sites in the analyzed genomes and consistency check or cross-species comparison of the predicted regulons (details are provided in Materials and Methods section; the strategy was also reviewed in [1]). For regulons with significantly different repertoire of target genes in the *Shewanella* spp., the above procedure was repeated starting at the third step in order to include novel candidate targets into the TFBS motif model and to revise the final gene content of the regulon.

For the *Shewanella* genomes, we performed regulon reconstruction for 41 TFs that are orthologous to previously characterized regulators (Table 1). The majority of these TFs have experimentally characterized orthologs in γ-proteobacteria from other lineages, such as *E. coli* (35 TFs) and/or *Pseudomonas* spp. (10 TFs), or had been previously studied in *S. oneidensis* (5 TFs) (Additional file 5). Among these regulators, there are 26 universal TFs, three strain-specific TFs and 13 TFs mosaically distributed in the *Shewanella* spp. The deduced TFBS motifs for 41 analyzed regulons in the *Shewanella* spp. were compared to previously known motifs for orthologous regulators in other γ-proteobacteria using the RegulonDB database for *E. coli*[35] and original publications for *Pseudomonas* spp. (Additional file 5). For three regulators with previously unknown binding sites (GlmR, HutC, and SdaR) we report, for the first time, the identity of their cognate TFBSs. The identified new motifs in *Shewanella* are conserved in upstream regions of known targets in *E. coli* (for SdaR) and *Pseudomonas* spp. (for GlmR and HutC) (data not shown). Two novel TFBS motifs (for AgaR and GcvA) in the *Shewanella* spp. are completely different from the respective motifs in *E. coli*. Five other TFBS motifs (for CueR, NhaR, PsrA, TrpR, and ZntR) in the *Shewanella* spp. are moderately different (3-4 mismatches in the conserved positions) from the known motifs of orthologous TFs previously described in *E. coli* and/or *Pseudomonas* spp. The remaining 31 *Shewanella* TFs appear to have binding motifs that are well conserved or only slightly different (1-2 mismatches in the conserved positions) from the motifs of their previously characterized orthologs.

#### Inference of novel regulons for metabolic pathways and chromosomal gene clusters

To identify novel regulons in the absence of experimental data, we used two types of potentially co-regulated gene sets: i) genes that constitute functional metabolic pathways (subsystems); and ii) genes derived from conserved gene neighborhoods that include a putative TF gene. To analyze metabolic subsystems and conserved chromosomal gene clusters projected across bacterial genomes we used the SEED database [36]. Each training set of potentially co-regulated operons was collected from 16 analyzed *Shewanella* genomes, and a collection of their upstream regions was used as an input for the motif-recognition program SignalX to predict a common DNA motif allowing a limited number of sequences to be ignored. At the next step, the *Shewanella* genomes were scanned with the constructed DNA motif to reveal the distribution of similar sites that were further verified by the consistency check procedure (reviewed in [1]). Finally, the genomic context of candidate co-regulated genes was used to attribute a potential TF to each novel regulon and associated DNA motif.

As a result, we inferred 41 novel regulons in *Shewanella* spp. including: i) 18 regulons for metabolic subsystems; and ii) 23 regulons for conserved chromosomal gene clusters (Table 2). The metabolic regulons from the first group control genes from the metabolic pathways of utilization of various carbohydrates, as well as formate, lactate, propionate, hydroxyproline/proline, tyrosine, and branched chain amino acids, and the purine biosynthesis pathway. All of these metabolic regulons except the purine regulon were assigned to a TF by a combination of different evidence types such as (i) positional clustering of target genes and TFs on the chromosome; ii) autoregulation of a TF by a cognate TFBS; iii) correlation in the phylogenetic pattern of co-occurrence of TFBSs and TFs in the genomes. Each of these novel TFs was functionally annotated in the SEED database (http://theseed.uchicago.edu) and tentatively named using an abbreviation of the target metabolic pathway/genes. Hereinafter we mark the new names by asterisks.

Most of the novel metabolic TFs represent non-orthologous replacement of previously known TFs that control similar metabolic pathways in other lineages. For example, the propionate catabolism in the Enterobacteria is activated by the Fis-family regulator PrpR, whereas in the *Shewanella* spp. it is predicted to be controlled by a GntR-family TF PrpR*. The proline utilization is controlled by the Lrp-family activator PutR in the *Vibrio* spp. [37], the AraC-family activator PruR in the *Pseudomonas* spp. [38], and the predicted GntR-family regulator HypR* in the *Shewanella* spp.. The homogentisate pathway of the tyrosine degradation is regulated by the IclR-type repressor HmgR in the *Pseudomonas* spp. [39], which is replaced by novel LysR-family regulator HmgR* in the *Shewanella* spp.. Similar non-orthologous replacements of regulators have been detected for ten different carbohydrate catabolic pathways [33] and the lactate utilization system in the *Shewanella* spp. [30]. A novel purine-pathway regulon (named PUR*) with hitherto unknown cognate TF was inferred in *Shewanella* instead of PurR regulon previously characterized in other γ-proteobacteria including *E. coli*[40] and missing in the *Shewanella* spp.. Two novel regulators PflR* and XltR* were predicted to control metabolic pathways of pyruvate to formate fermentation and xylitol catabolism, whose regulation have not yet been previously described in any bacteria.

Functional annotations of novel TF regulons that were deduced from the analysis of conserved gene clusters are largely hypothetical and incomplete. Most of them are local regulators controlling one or two target operons (Additional file 4). Two novel TF regulators from the Crp family, named DeoR* and PnuR*, control candidate phosphorylases and transporters likely involved in the nucleoside/nicotinamide ribose utilization. A novel AsnC-type regulator AzrR* controls the *azr-SO3586* operon, which encodes azoreductase and lactoylglutathione lyase that are likely involved in the superoxide stress protection. Novel regulator CalR* controls expression of the coniferyl aldehyde dehydrogenase *calB* that play a role in phytochemical aromatic compound utilization. Other inferred TF regulons appear to contain various hypothetical metabolite efflux transporters or flavocytochromes potentially involved in detoxification and undescribed respiratory processes, respectively.

#### Identification of regulons for RNA regulatory elements

We used known regulatory-RNA patterns from the Rfam database [41] to scan intergenic regions in 16 *Shewanella* genomes and analyzed the genomic context of candidate regulatory RNAs (Additional file 4).

Representatives of eight metabolite-responsive riboswitch families are scattered in most *Shewanella* genomes. The lysine, glycine, thiamine, cobalamin, riboflavin, and molybdenum cofactor riboswitches control genes for the respective amino acid / cofactor biosynthetic pathways and/or uptake transporters. The purine riboswitch controls adenosine deaminase and purine transporter. The riboswitch that binds second messenger cyclic di-GMP was found to control various subsets of genes in the *Shewanella* spp. including genes encoding extracellular proteins such as the chitin binding protein, chitinases, peptidases, and other hypothetical secreted proteins.

Six candidate attenuators that regulate operons responsible for the biosynthesis of branched chain amino acids, histidine, threonine, tryptophan, and phenylalanine in proteobacteria [42] are conserved in all analyzed *Shewanella* spp.

### Experimental validation of N-acetylglucosamine-responsive regulon NagR in *S. oneidensis* MR-1

A predicted transcriptional regulator NagR of the LacI family is a nonorthologous replacement of the NagC repressor from Enterobacteria. In addition to genes involved in Nag transport (*nagP* and *omp*^Nag^) and biochemical conversion (*nagK-nagB*^II^*-nagA*), the reconstructed NagR regulon contains auxiliary components that are likely involved in chemotaxis and hydrolysis of chitin and/or chitooligosaccharides (*mcp*^Nag^-*hex* and *cbp*). Experimental validation of the reconstructed NagR regulon in *S. oneidensis* MR-1 was performed by both *in vitro* and *in vivo* approaches. The *nagR* gene was cloned and overexpressed in *E. coli*, and the recombinant protein was purified by Ni^2+^-chelating chromatography. We used electrophoretic mobility shift assay to test specific DNA-binding of the purified NagR protein to its predicted operator sites in upstream regions of the *nagP* (*SO3503*), *nagK* (*SO3507*), *mcp*^Nag^ (*SO3510*), *omp*^Nag^ (*SO3514*) and *cbp* (*SO1072*) genes in *S. oneidensis* MR-1. The maximal shift of the *nagK* DNA fragment observed at 100 nM NagR was completely suppressed by the addition of 20 mM of *N-*acetylglucosamine, which was thus proven as a negative effector (Additional file 6A). Specific binding at 100 nM NagR protein was also confirmed for the other four tested DNA fragments. To confirm the negative regulatory effect of NagR on gene expression *in vivo*, the *S. oneidensis* ?*nagR* targeted deletion mutant was constructed and relative transcript levels of the predicted NagR target genes were analyzed by quantitative RT-PCR. Relative mRNA levels of the *nagP*, *nagK*, *mcp*^Nag^, *omp*^Nag^, and *cbp* genes were elevated 15-, 50-, 16- 11-, and 5-fold, respectively, in the ?*nagR* mutant compared to the wild-type strain when grown in the minimal medium supplied with lactate (Additional file 6B). These results confirm that NagR is a negative regulator of the chitin utilization genes that are de-repressed in response to N-acetylglucosamine.

## Discussion

### Conservation and variations in the regulatory network evolution

Conservation of 5738 regulatory interactions identified for all predicted members of the reconstructed regulons across the *Shewanella* genus is shown in Additional file 4. Overall, the regulatory systems of the *Shewanella* spp. appears out to be considerably variable within the genus and quite distinct from other previously studied γ-proteobacteria. The observed variations can be classified in three distinct types: (i) “regulon expansion” in the *Shewanella* compared to other lineages that can be ranged from additions of several regulon members to larger-scale shifts in the regulated metabolic pathways (e.g., HexR, PdhR, and TyrR regulons); (ii) “fuzzy regulons” when a regulon possess a conserved core and variable periphery within the *Shewanella* group (e.g., global regulons ArgR, Crp, Fur, NarP, and Fnr); (iii) “regulon loss or acquisition” when entire regulon (including all operons from a regulated pathway) is present only in some of the *Shewanella* species (e.g., for Dnr, ModE, BetI, and 17 regulons controlling various sugar utilization pathways [33]). Of course, this distinction is very schematic and in reality these types of behavior overlap. The mostly conserved regulatory interactions occur among TF regulons that are involved in the control of essential biosynthetic pathways (e.g., BirA, FabR, GlmR, IlvY, NrdR regulons), and universal stress responses (LexA and ZntR regulons).

To estimate the relative conservation of the predicted regulatory interactions in other lineages, we searched for orthologs of the putative regulon members in *E.coli* and compared the gene contents of the regulons reconstructed in the *Shewanella* and with orthologous regulons in *E.coli* captured in the RegulonDB database (Additional file 4). Similar analysis was performed for the *Shewanella* regulons characterized in the *Pseudomonas* spp. (but not in *E. coli*), including Dnr, GlmR, HexR, HutC, and PsrA (for references see Additional file 5). Among 468 cognate operons that belong to 42 studied regulons in the *Shewanella* spp., 138 operons (30%) have orthologous known targets in *E. coli* or *Pseudomonas*, 223 operons (~50%) lack orthologous operons, whereas the remaining 107 operons (~20%) have orthologous operons that are not under control of orthologous TFs in these species. Examples of impressive variations in the content of orthologous TF regulons in the *Shewanella* and *E. coli* are discussed below.

**Figure 3:**
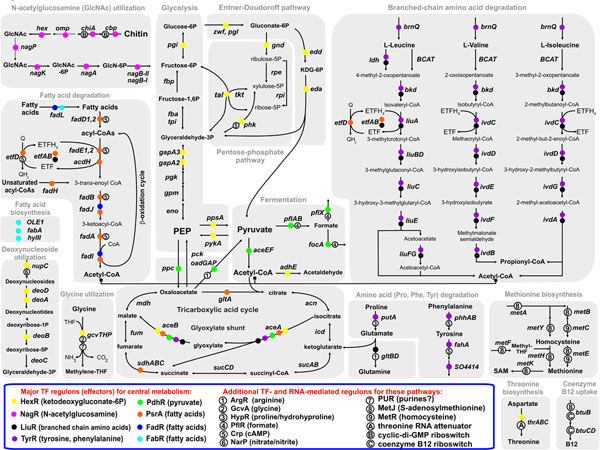
Metabolic context of the reconstructed regulons in *Shewanella* spp.

The comparison of the inferred regulons revealed striking differences in the strategies for regulation of the central carbohydrate and amino acid metabolism between the lineages comprising the *Shewanella* spp. and the Enterobacteria. In *E. coli*, two global regulators, FruR (fructose repressor/activator) and Crp (cAMP-responsive activator), control the central carbohydrate metabolism, whereas HexR (phospho-keto-deoxy-gluconate-responsive repressor) and PdhR (pyruvate repressor) are local regulators of glucose-6P dehydrogenase and pyruvate dehydrogenase, respectively. By contrast, the *Shewanella* spp. are predicted to use the HexR and PdhR regulators for the global control of the central carbohydrate metabolism and fermentation (Fig. 3). The FruR TF is absent in the *Shewanella* spp. that are not able to utilize fructose. The content and functional role of the Crp regulon is significantly different in the two lineages: the catabolism of carbohydrates and amino acids in the Enterobacteria, and the anaerobic respiration in the *Shewanella* spp. Most sugar catabolic pathways in the *Shewanella* spp. seem to be exclusively controlled by local sugar-responsive TFs that are often replaced by non-orthologous TFs (e.g., NagR vs. NagC for the N-acetylglucosamine utilization), and lack global co-regulation by Crp. Thus, the *Shewanella* spp. seem to lack many “feed-forward loops” that are characteristic for the regulation of sugar catabolism pathways in *E.coli* (when an operon is regulated by Crp and a local regulator that also is regulated by Crp) [43], thus may have a different strategy of sugar catabolism on mixed substrates.

Significant shifts in the regulon content were also identified for the TyrR, FadR, and FabR regulons (Fig. 3). In *E. coli*, the tyrosine- and phenylalanine-responsive regulator TyrR represses most aromatic amino acid biosynthetic enzymes and transporters encoded by multiple *aro* and *tyr* genes scattered on the chromosome, and activates the tyrosine transporter encoded by the *mtr* gene. In the *Shewanella* spp., we identified TyrR as a master regulator of the degradation pathways for various amino acids, including phenylalanine (*phhAB* operon), tyrosine (*fahA-maiA* operon), branched chain amino acids (*ldh*, *brnQ*, *liu*, *ivd*, and *bkd* operons), proline (*putA* gene), and oligopeptides (various peptidase genes), as well as some other pathways such as the glyoxylate shunt (*aceBA* operon), and the chorismate biosynthesis (*aroA* gene). These findings are in accordance with the previously established role of PhhR, a TyrR ortholog in *Pseudomonas* spp., as an activator for phenylalanine and tyrosine degradation genes [44]. The fatty acid degradation pathway in the *Shewanella* app. and many other γ-proteobacteria is controlled by PsrA, whereas in the Enterobacteria the analogous pathway is regulated by FadR [4]. The *Shewanella* spp. also have a significantly reduced in size FadR regulon, which retains only two operons shared with the orthologous regulon of *E. coli*, *fadIJ* and *fadL*[4]. Finally, the fatty acid biosynthesis regulon FabR has only one gene, *fabA*, which has conserved regulation in both *E. coli* and the *Shewanella* spp., whereas the remaining target genes were identified as a lineage-specific regulon extension.

### Interconnections between the predicted regulons in *Shewanella* spp

The collection of the inferred *Shewanella* regulons contains at least 30 regulons (for 24 TFs and 6 regulatory RNAs) that have at least one operon under simultaneous control of at least two regulators (Additional file 4). Most of the overlapping regulons control amino acid, fatty acid, nitrogen, and central carbohydrate metabolism (Fig. 3). The glyoxylate shunt operon *aceBA* controlled by five TFs is the most regulated operon in the current TRN model (see below). The glycine utilization operon *gcvTHP* was found to be controlled by the glycine-responsive regulator GcvA, the central carbohydrate regulator HexR, and the novel purine biosynthesis regulator PUR*. In the predicted regulons, 14 operons are under overlapping control of three regulons, whereas ~70 operons are co-regulated by two regulons. At least four regulatory cascades between various TFs were identified in the *Shewanella* spp.: LiuR for *tyrR*, NarP for *crp*, Crp for *hmgR*, and MetJ for *metR*, and only the latter cascade is conserved in *E. coli*.

**Figure 4:**
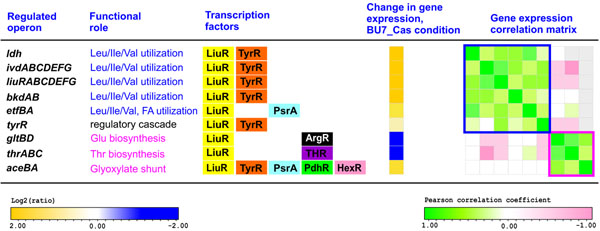
Multiple regulation of branched chain amino acid utilization regulon LiuR in *Shewanella oneidensis* MR-1

The reconstructed TRN provides insight into interplay between several different TFs controlling multiple genes from the LiuR regulon (Fig. 4). LiuR is a MerR-family repressor that controls the branched chain amino acid (Ile/Leu/Val) utilization in diverse proteobacteria [4]. In *Shewanella* spp., the predicted LiuR regulon was found to regulate Ile/Leu/Val operons (*ldh*, *liu*, *ivd*, and *bkd*) and was expanded by additional members involved in the biosynthesis of glutamate (*gltBD*) and threonine (*thrABC*), and the glyoxylate shunt (*aceBA*). Six out of nine LiuR-controlled operons are also regulated by the tyrosine/phenylalanine-responsive transcription factor TyrR [45]. Although TyrR in *E. coli* can act both as activator and repressor on its target genes, the mode of TyrR action on *Shewanella* targets is to be determined experimentally. Preliminary comparative analysis of relative positions of the TyrR- and LiuR-binding sites in *Shewanella* genomes (using multiple alignment of the promoter gene regions) suggests that TyrR probably acts as an activator for the *ldh*, *liu*, *ivd*, and *bkd* operons (data not shown). This supposition suggests that integrative effect of the LiuR and TyrR mediated control can be activation of their target genes in the simultaneous presence of Ile/Leu/Val and Tyr/Phe. Indeed, the expression data confirm strong up-regulation of the Ile/Leu/Val utilization and glyoxylate shunt genes in the presence of casein-derived mixture of amino acids (Fig. 4).

In contrast, two amino acid biosynthetic operons are down-regulated in the same condition. This observation can be explained by additional regulatory mechanisms found for each of these operons. The glutamate synthase *gltBD* is also controlled by ArgR, which is known to repress gene expression in the presence of arginine [46]. The threonine biosynthesis operon *thrABC* is also repressed by threonine availability using RNA attenuation mechanism [42].

Analysis of pairwise correlations for all LiuR-regulated genes based on ~200 microarray expression profiles available in the MicrobesOnLine database [47] allows us to identify two subregulons that have different gene expression patterns (Fig. 4). The first catabolic subregulon contains six operons, five of which are involved in the Ile/Leu/Val utilization, whereas the second subregulon has two biosynthetic operons and the glyoxylate shunt operon *aceBA*. The current TRN model has the largest number of regulatory interactions for the latter operon, which is controlled by five TFs including the Ile/Leu/Val repressor LiuR, the Tyr/Phe repressor/activator TyrR, the phospho-keto-deoxy-gluconate regulator HexR, the pyruvate repressor PdhR, and the fatty acid repressor PsrA. The glyoxylate shunt pathway plays a central metabolic role by providing intermediates required for amino acid biosynthesis, and being involved in the utilization of acetyl-CoA, a common product of the Ile/Leu/Val amino acids, fatty acids and carbohydrate degradation pathways [48].

### Conclusions and future perspectives

By applying the comparative genomics approach, we tentatively defined the first reference collection of transcriptional regulons in 16 *Shewanella* genomes comprised of 82 orthologous groups of TFs, ~7,300 TF-binding sites (~450 per genome), and 258 RNA regulatory motifs from 14 families. The resulting regulatory network contains ~600 regulated genes per genome that are mostly involved in the central metabolism, production of energy and biomass, metal ion homeostasis and stress responses. Although some diversity of the predicted regulons was observed within the *Shewanella* genus, the most significant diversification and adaptive evolution of TRNs were revealed by comparison with the established TRN in *E. coli* and related Enterobacteria. These differences are mostly attributed to: i) lineage specific regulon expansion and contraction for orthologous TFs that use conserved TFBS consensus motifs, and ii) involvement of non-orthologous TFs to control physiologically equivalent metabolic pathways in the two lineages of γ-proteobacteria.

The reconstructed regulons in *S. oneidensis* MR-1 are supported by available microarray expression data for the *fur*, *crp*, and *etrA* (*fnr*) knockout strains [25,26,28,29], as well as for the wild type strain grown on various carbon sources (inosine, N-acetylglucosamine, amino acids, lactate, and pyruvate) [20]. Preliminary analysis of correlations in expression patterns of genes from predicted regulons was useful for the interpretation of the reconstructed TRN, as illustrated by the LiuR regulon example. We are currently expanding this approach to other data. Targeted experimental validation of eight novel regulons for central carbohydrate and amino acid metabolism in *S. oneidensis* MR-1 is currently underway. Previously we have characterized *in vitro* the novel NAD metabolism regulon NrtR [11] and in this work we present *in vivo* and *in vitro* validation of N-acetylglucosamine utilization regulon NagR. Combined *in vivo* and *in vitro* experimental validation of the global carbohydrate metabolism regulon HexR and the assessment of its physiological role in *Shewanella* will be published elsewhere.

This work demonstrates the power of the comparative genomics approach in application to the reconstruction of transcriptional regulons in poorly studied groups of related bacteria. The reference set of the *Shewanella* regulons is the first taxonomy-wide collection of regulons obtained by this approach. It can be assessed in the RegPrecise database [34]. We anticipate a fast growth of taxonomy-wide regulon collections for other lineages in the near future. Regulatory interactions from the reconstructed regulons will provide an additional regulatory constrains for the recently published metabolic model of *S. oneidensis* MR-1 [22], allowing one to build an integrated model of metabolism and regulation. Such integrated model can be used for phenotype prediction, functional gene assignment and understanding of organism ecology. Finally, the reconstructed regulons were useful for the genome context-based prediction of novel functions of enzymes and transporters in previously uncharacterized carbohydrate utilization pathways in *Shewanella* spp. [33]

## Methods

### Bioinformatics methods for regulon reconstruction and used databases

The *Shewanella* spp. genomes were downloaded from the Genbank [49] (Fig. 1). The set of predicted DNA-binding TFs was extracted from the DBD database [50]. The *locus_tag* gene identifiers are used throughout. Orthologous proteins in 16 *Shewanella* genomes were defined in the previous work by the best bidirectional hits criterion [32]. Orthologous groups in *Shewanella* were named by either a common name of characterized protein, a novel name for proteins functionally annotated in this study, or by a *locus_tag* from *S. oneidensis* genome for uncharacterized proteins. Orthologs between proteins from different taxonomic groups (e.g. *Shewanella* and other γ-proteobacteria) were defined as bidirectional best hits with 30% of identity threshold using the Smith-Waterman algorithm implemented in the GenomeExplorer program [51]. In dubious cases orthologs were confirmed by construction of phylogenetic trees and comparative analysis of gene neighborhoods using the MicrobesOnline tree browse tool [47]. Functional gene assignments and metabolic subsystem analysis were performed using the SEED annotation/analysis tool http://theseed.uchicago.edu/FIG/index.cgi, which combines protein similarity search, positional gene clustering, and phylogenetic profiling of genes [36]. In addition, the InterPro [52], and PFAM [53] databases were used to verify protein functional and structural annotations.

For *de novo* identification of a candidate regulatory motif in the training set of potential upstream regions of genes (intergenic regions up to 350 bp) we used a simple iterative procedure DNA motif detection procedure implemented in the program SignalX [54]. Weak palindromes were selected in each region. Each palindrome was compared to all other palindromes, and the palindromes most similar to the initial one were used to make a profile. The candidate site score was defined as the sum of the respective positional nucleotide weights [7]. These profiles were used to scan the set of palindromes again, and the procedure was iterated until convergence. Thus a set of PWM profiles was constructed. A profile with largest information content was used as the recognition rule [55]. Each genome encoding the studied TF was scanned with the constructed motif profile using the GenomeExplorer software [51] and genes with candidate regulatory sites in the upstream regions were selected. The threshold for the site search was defined as the lowest score observed in the training set. Among new candidate members of a regulon, only genes having candidate sites conserved in at least two other genomes were retained for further analysis. We also included new candidate regulon members that are functionally related to the established regulon members. Additional and more detailed description of various scenario for regulon reconstruction using comparative genomics was reviewed in [1]. Analysis of large regulons (Fur, Crp, Fnr, NarP, LexA) was carried out using the web-based tool RegPredict allowing the comparative genomics-based regulon inference http://regpredict.lbl.gov[56]. The details of reconstructed regulons were captured and displayed in our recently developed database RegPrecise http://regprecise.lbl.gov[34]. For identification of RNA regulatory motif sequences we scanned complete genomes using tools and profiles available from the Rfam database [41]. Calculation of the Pearson coefficient for the LiuR-regulated genes was done by tools available at the MicrobesOnLine resource [47].

### Experimental methods for regulon validation

The *nagR* (SO3516) gene cloned at a pET-derived vector containing the T7 promoter and His_6_ tag [57] was kindly provided by Frank Collart (Argonne National Laboratory, IL).

***Protein purification****.* Recombinant proteins of *nagR* (SO3516) from *S. oneidensis* MR-1 was overexpressed as N-terminal fusion with a His_6_ tag in *E. coli* strain BL21/DE3. Cells were grown on LB media to OD_600_ = 0.8 at 37°C, induced by 0.2mM IPTG, and harvested after 12 h shaking at 20°C. Protein purification was performed using rapid Ni-NTA agarose minicolumn protocol as described [58]. Briefly, harvested cells were resuspended in 20 mM HEPES buffer pH 7 containing 100 mM NaCl, 0.03% Brij 35, and 2 mM β-mercaptoethanol supplemented with 2 mM phenylmethylsulfonyl fluoride and a protease inhibitor cocktail (Sigma-Aldrich). Lysozyme was added to 1 mg/mL, and the cells were lyzed by freezing-thawing followed by sonication. After centrifugation at 18,000 rpm, the Tris-HCl buffer (pH 8) was added to the supernatant (50 mM, final concentration), and it was loaded onto a Ni-NTA agarose column (0.2 ml). After washing with the starting buffer containing 1 M NaCl and 0.3% Brij-35, bound proteins were eluted with 0.3 ml of the starting buffer containing 250 mM imidazole. Protein size, expression level, distribution between soluble and insoluble forms, and extent of purification were monitored by SDS-PAGE.

***qPCR.*** In-frame deletion mutagenesis of or *nagR* (*SO3516*) was performed using previously published method [33]. Genomic RNA was isolated from *S. oneidensis* MR-1 and ∆*nagR* cells grown in minimal medium supplied with lactate and collected at O.D.(600) of 0.52 using the RNA purification kit from Promega (Madison, WI). Reverse transcription of total RNA was performed with random primers using iScript cDNA synthesis kit from BIO-RAD (Hercules, CA), following kits instructions. qPCR was performed using SYBR GreenER qPCR SupeMix Universal kit from Invitrogene (Carlsbad, CA). Transcript levels of the *nagP* (SO3503), *nagK* (*SO3507*), *mcp*^Nag^ (*SO3510*), *omp*^Nag^ (*SO3514*), *cbp* (*SO1072*), *SO0854*, and *zwf* (*SO2489*, used as a negative control) genes were measured and the results were normalized to the expression level of 16S mRNA. Fold change was calculated by the 2^-∆CT^ method [59] as a ratio of normalized mRNA levels in ∆*nagR* mutant and wild-type MR-1 strains.

***DNA-binding assay.*** Interaction of purified recombinant protein NagR (SO3516) from *S. oneidensis* MR-1 with their cognate DNA motifs was assessed by EMSA technique using the following dsDNA segments obtained by PCR amplification or by custom synthesis of both complementary oligonucleotides (IDT, San Diego, CA), annealing and purification. One of the primers was 5′-biotinylated (IDT). By using *S. oneidensis* MR-1 DNA as the template, we amplified DNA fragments from the following upstream gene regions: SO1072 (89 bp), SO3507 (69 bp), SO3510 (64 bp), SO3514 (69 bp), SO3503 (62 bp), SO0854 (67 bp). For EMSA, the biotin-labeled DNA (0.1 or 1 nM) was incubated with the increasing amount of purified NagR (0-100 nM) in a total volume of 20 μl. The binding buffer contains Tris-HCl 20mM, KCl 150mM, MgCl2 5mM, DTT 1mM, EDTA 1mM, 0.05% NP-40, 2.5% glycerol. The poly(dI-dC) (Sigma) was added as nonspecific competitor DNA at ~10^4^-fold molar excess over labeled target DNA to reduce nonspecific binding. After 25 min incubation at room temperature, the reaction mixtures were separated by electrophoresis on a 5% native polyacrylamide gel in 0.5 × Tris-borate-EDTA for 90 min at 90V, at 4°C. The gel was transferred by electrophoresis (30 min, at 380 mA) onto a nylon membrane (Pierce, Rockford, Ill.) and fixed by UV cross-linking. Biotin-labeled DNA was detected with the LightShift Chemiluminescent EMSA kit (Pierce, Rockford, Ill.), as recommended by the manufacturer. The effect of *N-*acetyl-glucosamine on NagR-DNA binding was tested by addition of 20 mM of *N-*acetylglucosamine to the incubation mixture.

## List of abbreviations used

TF: transcription factor; TFBS: transcription factor-binding site; TRN: transcriptional regulatory network.

## Authors’ contributions

DARo, ALO and MSG conceived and supervised the research, and wrote the manuscript. DARo, IAR, DARa, AEK, EAP, AVG, ONL, GYK performed comparative genomic analysis to infer novel transcription factor regulons. MDK identified riboswitches. PSN developed RegPredict tool and RegPrecise database, performed propagation of regulons. DARo and PSN performed correlation analysis using expression data. EDS and RO performed computational similarity searches and gene annotation in the SEED database. MFR produced manually annotated table of orthologs and provided targeted gene knockout strains in *Shewanella*. XL carried out validation experiments for NagR regulon. JKF, ID and APA contributed to the development of the manuscript and design of the study. All authors read and approved the final manuscript.

## Competing interests

The authors declare that they have no competing interests.

## Supplementary Material

Additional file 1 Rodionov_AF1.xls - Repertoire of DNA-binding transcription factors identified in 16 genomes of *Shewanella* spp. Click here for file

Additional file 2 Rodionov_AF2.xls - Transcription factors universally conserved in *Shewanella* spp. Click here for file

Additional file 3 Rodionov_AF3.xls - Functional classifiaction of 73 transcription factors conserved between *E. coli* and 16 *Shewanella* strains. Click here for file

Additional file 4 Rodionov_AF4.xls - Functional content and conservation of reconstructed TF and RNA regulons in *Shewanella* genomes. Click here for file

Additional file 5 Rodionov_AF5.pdf - Comparison of predicted TFBS motifs in *Shewanella* spp. to the previously characterized orthologous regulators in model species. Click here for file

Additional file 6 Rodionov_AF6.pdf - Experimental validation of NagR regulon in *S. oneidensis* MR-1. (A) Electrophoretic mobility shift assays to assess NagR protein binding to their predicted DNA operators; (B) Differential regulation of NagR controlled genes determined by quantitative qPCR. Click here for file
