# Supplementary material for: Comparative genomic reconstruction of transcriptional networks controlling central metabolism in the Shewanella genus
Source: BMC Genomics. 2011 Jun 15;12(Suppl 1):S3. doi: 10.1186/1471-2164-12-S1-S3 (PMC3223726; doi:10.1186/1471-2164-12-S1-S3)
Supplement: Additional file 5 — Rodionov_AF5.pdf - Comparison of predicted TFBS motifs in Shewanella spp. to the previously characterized orthologous regulators in model species. [file 1471-2164-12-S1-S3-S5.pdf]

Additional file 5. **Comparison of predicted TFBS motifs in *Shewanella* spp. to the previously characterized orthologous regulators in model species.**

TFs	Predicted TFBS motif in *Shewanella*		Previously known motif for orthologous TFs in other species			Experimental data source	
	Motif sequence LOGO[1]	NS[2]	Motif sequence LOGO[1]	NS[2]	TFBSs data source[3]	Model species[4]	References[5]
**AgaR**	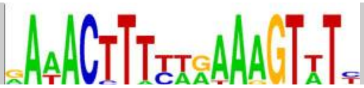	8 (4)	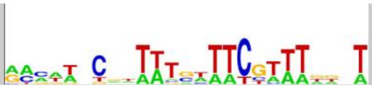	11 (1)	*E. coli* [RegulonDB]	*E. coli*	[1]
**ArgR**	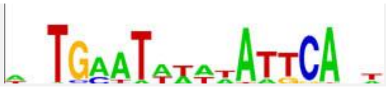	510 (16)	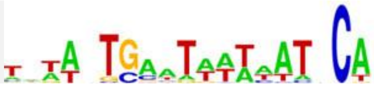	27 (1)	*E. coli* [RegulonDB]	*E. coli*	[2, 3]
**BetI**	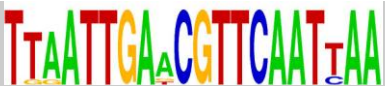	9 (8)	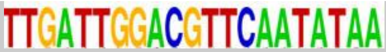	1 (1)	*E. coli* [4]	*E. coli*	[4]
**BirA**	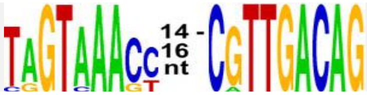	16 (16)	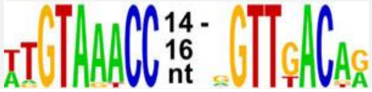	13 (13)	γ-proteobacteria [5]	*E. coli*	[6]
**Crp**	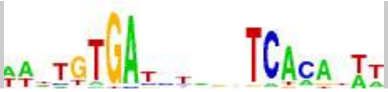	1262 (16)	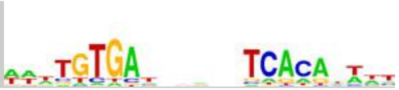	230 (1)	*E. coli* [RegulonDB]	*E. coli*, *S. oneidensis*	[7-10]
**CueR**	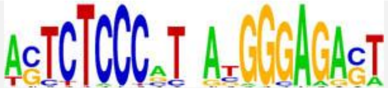	26 (14)	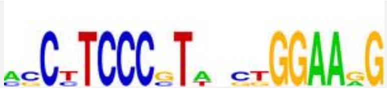	3 (1)	*E. coli* [RegulonDB]	*E. coli*, *S. typhimurium*, *P. fluorescens*	[11, 12]
**Dnr**	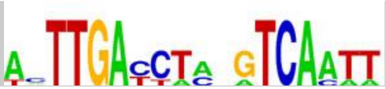	5 (3)	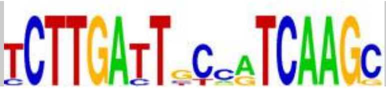	6 (3)	*Pseudomonas* spp. [13]	*Pseudomonas* spp.	[14]
**ExuR**	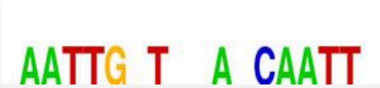	2 (1)	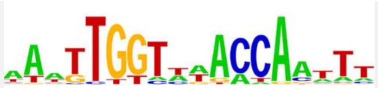	20 (3)	Enterobacteria [15]	*E. coli*, *E. chrysanthemi*	[16, 17]
**FabR**	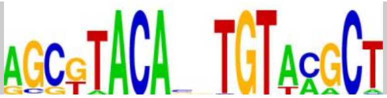	117 (16)	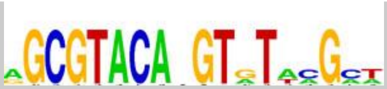	3 (1)	*E. coli* [18]	*E. coli*	[19]
**FadR**	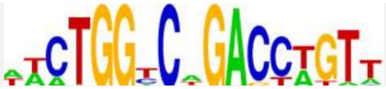	72 (16)	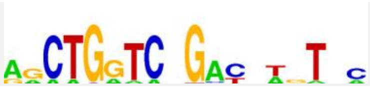	12 (1)	*E. coli* [RegulonDB]	*E. coli*	[20]
**Fnr**	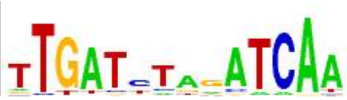	820 (16)	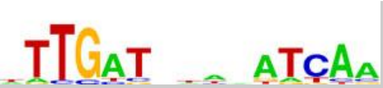	83 (1)	*E. coli* [RegulonDB]	*E. coli*, *S. oneidensis* (EtrA)	[21, 22]
**Fur**	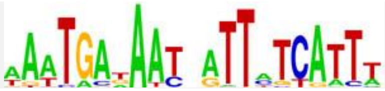	496 (16)	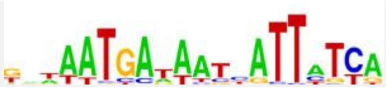	71 (1)	*E. coli* [RegulonDB]	*E. coli*, *S. oneidensis*, *P. aeruginosa*,	[10, 23-27]
**GalR**	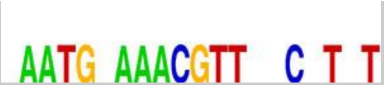	2 (1)	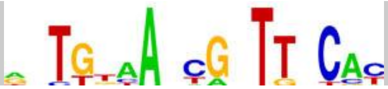	12 (1)	*E. coli* [RegulonDB]	*E. coli*	[28]
**GcvA**	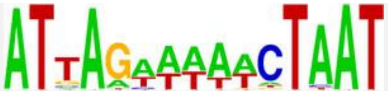	104 (16)	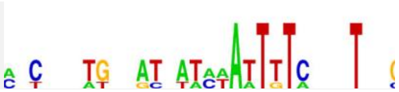	4 (1)	*E. coli* [RegulonDB]	*E. coli*	[29]

**GlmR**	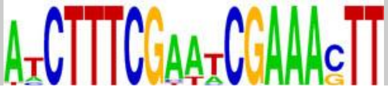	16 (16)	TFBS motif was unknown	-	-	*P. aeruginosa*	[30]
**GntR**	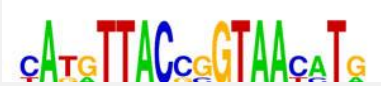	4 (1)	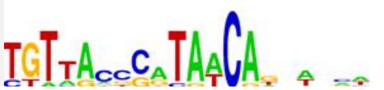	10 (1)	*E. coli* [RegulonDB]	*E. coli*	[31]
**HexR**	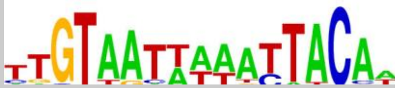	277 (16)	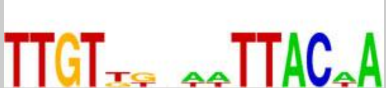	3 (1)	*P. putida* [32]	*P. putida*	[32]
**HutC**	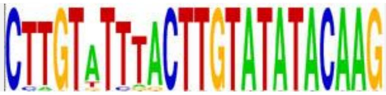	32 (16)	TFBS motif was unknown	-	-	*P. fluorescens*	[33]
**IlvY**	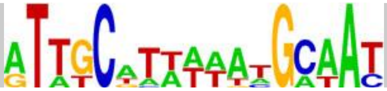	64 (16)	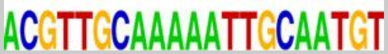	1 (1)	*E. coli* [34]	*E. coli*	[34]
**IscR**	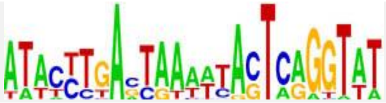	85 (16)	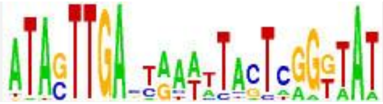	22 (5)	γ-proteobacteria [RegPrecise]	*E. coli*	[35]
**LexA**	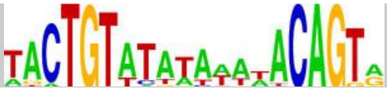	190 (16)	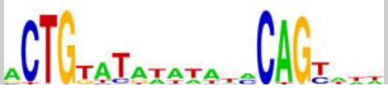	39 (1)	*E. coli* [RegulonDB]	*E. coli*, *P. putida*	[36, 37]
**MetJ**	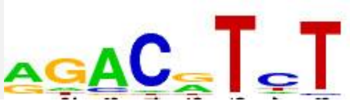	148 (16)	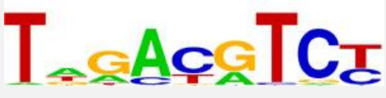	27 (1)	*E. coli* [RegulonDB]	*E. coli*	[38]
**MetR**	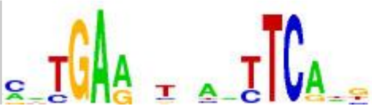	106 (16)	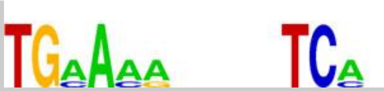	4 (1)	*E. coli* [RegulonDB]	*E. coli*	[39]
**ModE**	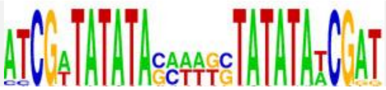	10 (5)	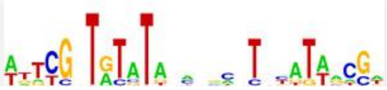	7 (1)	*E. coli* [RegulonDB]	*E. coli*	[40, 41]
**NanR**	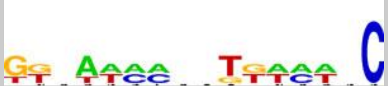	4 (1)	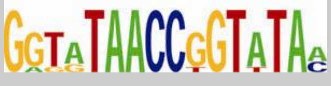	15 (5)	γ-proteobacteria [RegPrecise]	*E. coli*	[42]
**NarP**	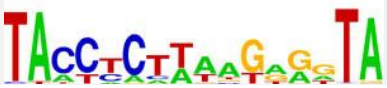	235 (16)	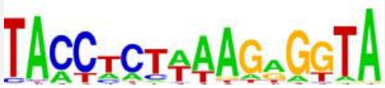	35 (10)	γ-proteobacteria [43]	*E. coli*	[22, 43]
**NhaR**	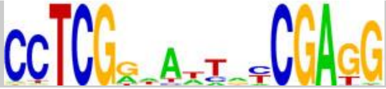	33 (16)	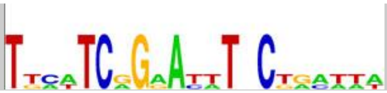	3 (1)	*E. coli* [RegulonDB]	*E. coli*	[44]
**NikR**	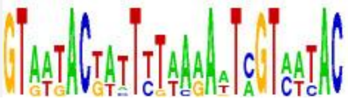	8 (3)	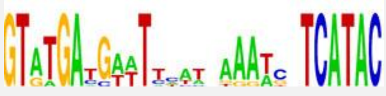	8 (8)	γ-proteobacteria [45]	*E. coli*	[46]
**NorR**	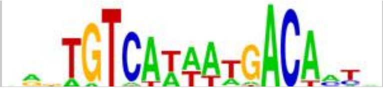	67 (13)	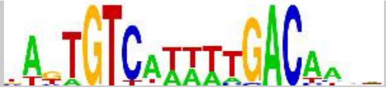	50 (14)	γ-proteobacteria [13]	*E. coli*	[47]

**NrdR**	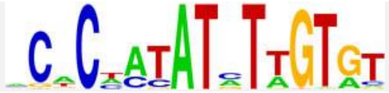	64 (16)	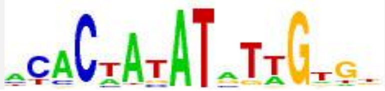	151 (35)	γ-proteobacteria [RegPrecise] [48]	*E. coli*	[49]
**NrtR**	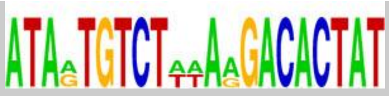	6 (3)	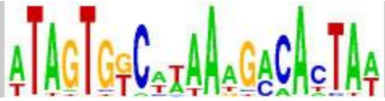	14 (6)	γ-proteobacteria [RegPrecise]	*S. oneidensis*	[50]
**NsrR**	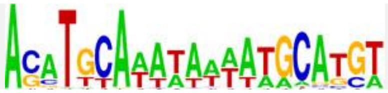	50 (16)	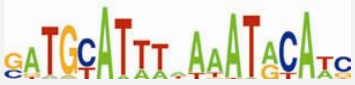	50 (12)	γ-proteobacteria [13]	*E. coli*	[51]
**NtrC**	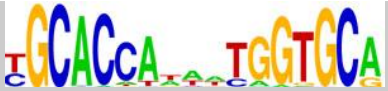	81 (16)	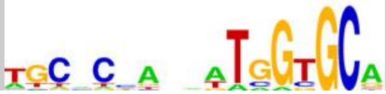	17 (1)	*E. coli* [RegulonDB]	*E. coli, P. putida*	[52]
**PdhR**	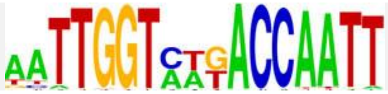	86 (16)	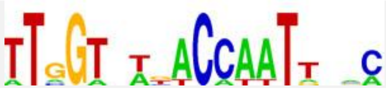	7 (1)	*E. coli* [RegulonDB]	*E. coli*	[53]
**PsrA**	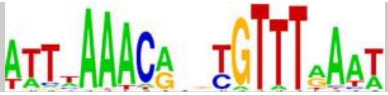	281 (16)	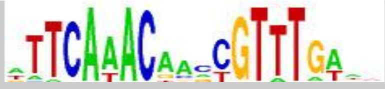	46 (5)	*Pseudomonas* spp. [RegPrecise]	*P. aeruginosa*	[54, 55]
**RbsR**	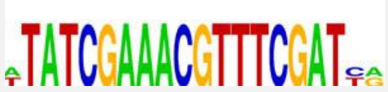	4 (2)	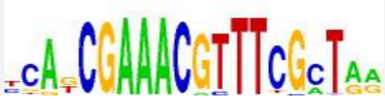	72 (48)	γ-proteobacteria [RegPrecise]	*E. coli*	[56]
**SdaR**	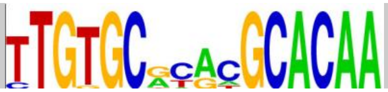	14 (14)	TFBS motif was unknown	-	-	*E. coli*	[57]
**SoxR**	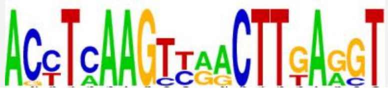	11 (5)	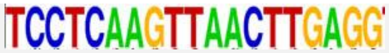	1 (1)	*E. coli* [58]	*E. coli, P. putida*	[58, 59]
**TorR**	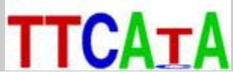	33 (13)	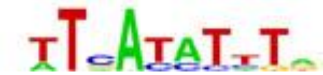	8 (1)	*E. coli* [RegulonDB]	*E. coli, S. oneidensis*	[60, 61]
**TrpR**	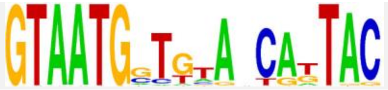	30 (16)	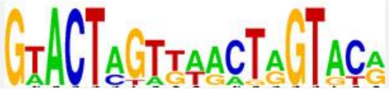	10 (1)	*E. coli* [RegulonDB]	*E. coli*	[62]
**TyrR**	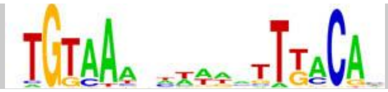	259 (16)	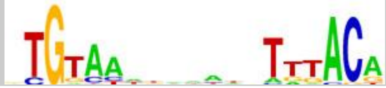	19 (1)	*E. coli* [RegulonDB]	*E. coli*	[63-65]
**ZntR**	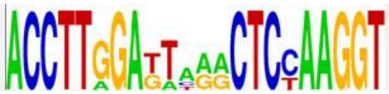	16 (16)	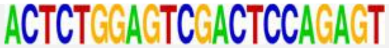	1 (1)	*E. coli* [66]	*E. coli*	[66]

[1] Sequence LOGOs were drawn using WebLogo package (http://weblogo.berkeley.edu/logo.cgi) [67].

[2] NS, number of TFBSs used to construct sequence LOGO. Number of genomes containing TFBSs used for LOGO construction is given in parenthesis.

[3] Organisms used to collect TFBSs and construct consensus motif. TFBS data sources, that are the RegulonDB database [68], the RegPrecise database [69] or the original publication, are shown in square brackets.

[4] Organism abbreviations: *E. coli, Escherichia coli; P. aeruginosa, Pseudomonas aeruginosa, P. putida, Pseudomonas putida; P. fluorescens, Pseudomonas fluorescens; S. typhimurium, Salmonella typhimurium; E. chrysantmeni, Erwinia chrysanthemi; S. oneidensis, Shewanella oneidensis;*

[5] References

1. Ray WK, Larson TJ: **Application of AgaR repressor and dominant repressor variants for verification of a gene cluster involved in N-acetylgalactosamine metabolism in Escherichia coli K-12.** *Mol Microbiol* 2004, **51:**813-826.
2. Caldara M, Charlier D, Cunin R: **The arginine regulon of Escherichia coli: whole-system transcriptome analysis discovers new genes and provides an integrated view of arginine regulation.** *Microbiology* 2006, **152:**3343-3354.
3. Caldara M, Minh PN, Bostoen S, Massant J, Charlier D: **ArgR-dependent repression of arginine and histidine transport genes in Escherichia coli K-12.** *J Mol Biol* 2007, **373:**251-267.
4. Rkenes TP, Lamark T, Strom AR: **DNA-binding properties of the BetI repressor protein of Escherichia coli: the inducer choline stimulates BetI-DNA complex formation.** *J Bacteriol* 1996, **178:**1663-1670.
5. Rodionov DA, Mironov AA, Gelfand MS: **Conservation of the biotin regulon and the BirA regulatory signal in Eubacteria and Archaea.** *Genome Res* 2002, **12:**1507-1516.
6. Brown PH, Cronan JE, Grotli M, Beckett D: **The biotin repressor: modulation of allostery by corepressor analogs.** *J Mol Biol* 2004, **337:**857-869.
7. Murphy JN, Durbin KJ, Saltikov CW: **Functional roles of arcA, etrA, cyclic AMP (cAMP)-cAMP receptor protein, and cya in the arsenate respiration pathway in Shewanella sp. strain ANA-3.** *J Bacteriol* 2009, **191:**1035-1043.
8. Saffarini DA, Schultz R, Beliaev A: **Involvement of cyclic AMP (cAMP) and cAMP receptor protein in anaerobic respiration of Shewanella oneidensis.** *J Bacteriol* 2003, **185:**3668-3671.
9. Gosset G, Zhang Z, Nayyar S, Cuevas WA, Saier MH, Jr.: **Transcriptome analysis of Crp-dependent catabolite control of gene expression in Escherichia coli.** *J Bacteriol* 2004, **186:**3516-3524.
10. Zhang Z, Gosset G, Barabote R, Gonzalez CS, Cuevas WA, Saier MH, Jr.: **Functional interactions between the carbon and iron utilization regulators, Crp and Fur, in Escherichia coli.** *J Bacteriol* 2005, **187:**980-990.
11. Espariz M, Checa SK, Audero ME, Pontel LB, Soncini FC: **Dissecting the Salmonella response to copper.** *Microbiology* 2007, **153:**2989-2997.
12. Zhang XX, Rainey PB: **Regulation of copper homeostasis in Pseudomonas fluorescens SBW25.** *Environ Microbiol* 2008, **10:**3284-3294.
13. Rodionov DA, Dubchak IL, Arkin AP, Alm EJ, Gelfand MS: **Dissimilatory metabolism of nitrogen oxides in bacteria: comparative reconstruction of transcriptional networks.** *PLoS Comput Biol* 2005, **1:**e55.
14. Castiglione N, Rinaldo S, Giardina G, Cutruzzola F: **The transcription factor DNR from Pseudomonas aeruginosa specifically requires nitric oxide and haem for the activation of a target promoter in Escherichia coli.** *Microbiology* 2009, **155:**2838-2844.
15. Rodionov DA, Mironov AA, Rakhmaninova AB, Gelfand MS: **Transcriptional regulation of transport and utilization systems for hexuronides, hexuronates and hexonates in gamma purple bacteria.** *Mol Microbiol* 2000, **38:**673-683.
16. Valmeekam V, Loh YL, San Francisco MJ: **Control of exuT activity for galacturonate transport by the negative regulator ExuR in Erwinia chrysanthemi EC16.** *Mol Plant Microbe Interact* 2001, **14:**816-820.
17. Bates Utz C, Nguyen AB, Smalley DJ, Anderson AB, Conway T: **GntP is the Escherichia coli Fructuronic acid transporter and belongs to the UxuR regulon.** *J Bacteriol* 2004, **186:**7690-7696.
18. McCue L, Thompson W, Carmack C, Ryan MP, Liu JS, Derbyshire V, Lawrence CE: **Phylogenetic footprinting of transcription factor binding sites in proteobacterial genomes.** *Nucleic Acids Res* 2001, **29:**774-782.

19. Zhu K, Zhang YM, Rock CO: **Transcriptional regulation of membrane lipid homeostasis in Escherichia coli.** *J Biol Chem* 2009, **284:**34880-34888.
20. Fujita Y, Matsuoka H, Hirooka K: **Regulation of fatty acid metabolism in bacteria.** *Mol Microbiol* 2007, **66:**829-839.
21. Beliaev AS, Thompson DK, Fields MW, Wu L, Lies DP, Nealson KH, Zhou J: **Microarray transcription profiling of a Shewanella oneidensis etrA mutant.** *J Bacteriol* 2002, **184:**4612-4616.
22. Constantinidou C, Hobman JL, Griffiths L, Patel MD, Penn CW, Cole JA, Overton TW: **A reassessment of the FNR regulon and transcriptomic analysis of the effects of nitrate, nitrite, NarXL, and NarQP as Escherichia coli K12 adapts from aerobic to anaerobic growth.** *J Biol Chem* 2006, **281:**4802-4815.
23. Cornelis P, Matthijs S, Van Oeffelen L: **Iron uptake regulation in Pseudomonas aeruginosa.** *Biometals* 2009, **22:**15-22.
24. Thompson DK, Beliaev AS, Giometti CS, Tollaksen SL, Khare T, Lies DP, Nealson KH, Lim H, Yates J, 3rd, Brandt CC, et al: **Transcriptional and proteomic analysis of a ferric uptake regulator (fur) mutant of Shewanella oneidensis: possible involvement of fur in energy metabolism, transcriptional regulation, and oxidative stress.** *Appl Environ Microbiol* 2002, **68:**881-892.
25. Wan XF, Verberkmoes NC, McCue LA, Stanek D, Connelly H, Hauser LJ, Wu L, Liu X, Yan T, Leaphart A, et al: **Transcriptomic and proteomic characterization of the Fur modulon in the metal-reducing bacterium Shewanella oneidensis.** *J Bacteriol* 2004, **186:**8385-8400.
26. Yang Y, Harris DP, Luo F, Wu L, Parsons AB, Palumbo AV, Zhou J: **Characterization of the Shewanella oneidensis Fur gene: roles in iron and acid tolerance response.** *BMC Genomics* 2008, **9 Suppl 1:**S11.
27. Yang Y, Harris DP, Luo F, Xiong W, Joachimiak M, Wu L, Dehal P, Jacobsen J, Yang Z, Palumbo AV, et al: **Snapshot of iron response in Shewanella oneidensis by gene network reconstruction.** *BMC Genomics* 2009, **10:**131.
28. Geanacopoulos M, Adhya S: **Functional characterization of roles of GalR and GalS as regulators of the gal regulon.** *J Bacteriol* 1997, **179:**228-234.
29. Wilson RL, Urbanowski ML, Stauffer GV: **DNA binding sites of the LysR-type regulator GcvA in the gcv and gcvA control regions of Escherichia coli.** *J Bacteriol* 1995, **177:**4940-4946.
30. Ramos-Aires J, Plesiat P, Kocjancic-Curty L, Kohler T: **Selection of an antibiotic-hypersusceptible mutant of Pseudomonas aeruginosa: identification of the GlmR transcriptional regulator.** *Antimicrob Agents Chemother* 2004, **48:**843-851.
31. Peekhaus N, Conway T: **Positive and negative transcriptional regulation of the Escherichia coli gluconate regulon gene gntT by GntR and the cyclic AMP (cAMP)-cAMP receptor protein complex.** *J Bacteriol* 1998, **180:**1777-1785.
32. Daddaoua A, Krell T, Ramos JL: **Regulation of glucose metabolism in Pseudomonas: the phosphorylative branch and entner-doudoroff enzymes are regulated by a repressor containing a sugar isomerase domain.** *J Biol Chem* 2009, **284:**21360-21368.
33. Zhang XX, Rainey PB: **Genetic analysis of the histidine utilization (hut) genes in Pseudomonas fluorescens SBW25.** *Genetics* 2007, **176:**2165-2176.
34. Rhee KY, Senear DF, Hatfield GW: **Activation of gene expression by a ligand-induced conformational change of a protein-DNA complex.** *J Biol Chem* 1998, **273:**11257-11266.
35. Giel JL, Rodionov D, Liu M, Blattner FR, Kiley PJ: **IscR-dependent gene expression links iron-sulphur cluster assembly to the control of O2-regulated genes in Escherichia coli.** *Mol Microbiol* 2006, **60:**1058-1075.

36. Abella M, Campoy S, Erill I, Rojo F, Barbe J: **Cohabitation of two different lexA regulons in Pseudomonas putida.** *J Bacteriol* 2007, **189:**8855-8862.
37. Kelley WL: **Lex marks the spot: the virulent side of SOS and a closer look at the LexA regulon.** *Mol Microbiol* 2006, **62:**1228-1238.
38. He YY, Stockley PG, Gold L: **In vitro evolution of the DNA binding sites of Escherichia coli methionine repressor, MetJ.** *J Mol Biol* 1996, **255:**55-66.
39. Lorenz E, Stauffer GV: **Characterization of the MetR binding sites for the glyA gene of Escherichia coli.** *J Bacteriol* 1995, **177:**4113-4120.
40. McNicholas PM, Gunsalus RP: **The molybdate-responsive Escherichia coli ModE transcriptional regulator coordinates periplasmic nitrate reductase (napFDAGHBC) operon expression with nitrate and molybdate availability.** *J Bacteriol* 2002, **184:**3253-3259.
41. Studholme DJ, Pau RN: **A DNA element recognised by the molybdenum-responsive transcription factor ModE is conserved in Proteobacteria, green sulphur bacteria and Archaea.** *BMC Microbiol* 2003, **3:**24.
42. Kalivoda KA, Steenbergen SM, Vimr ER, Plumbridge J: **Regulation of sialic acid catabolism by the DNA binding protein NanR in Escherichia coli.** *J Bacteriol* 2003, **185:**4806-4815.
43. Ravcheev DA, Gerasimova AV, Mironov AA, Gelfand MS: **Comparative genomic analysis of regulation of anaerobic respiration in ten genomes from three families of gamma-proteobacteria (Enterobacteriaceae, Pasteurellaceae, Vibrionaceae).** *BMC Genomics* 2007, **8:**54.
44. Carmel O, Rahav-Manor O, Dover N, Shaanan B, Padan E: **The Na+-specific interaction between the LysR-type regulator, NhaR, and the nhaA gene encoding the Na+/H+ antiporter of Escherichia coli.** *EMBO J* 1997, **16:**5922-5929.
45. Rodionov DA, Hebbeln P, Gelfand MS, Eitinger T: **Comparative and functional genomic analysis of prokaryotic nickel and cobalt uptake transporters: evidence for a novel group of ATP-binding cassette transporters.** *J Bacteriol* 2006, **188:**317-327.
46. Schreiter ER, Wang SC, Zamble DB, Drennan CL: **NikR-operator complex structure and the mechanism of repressor activation by metal ions.** *Proc Natl Acad Sci U S A* 2006, **103:**13676-13681.
47. D'Autreaux B, Tucker N, Spiro S, Dixon R: **Characterization of the nitric oxide-reactive transcriptional activator NorR.** *Methods Enzymol* 2008, **437:**235-251.
48. Rodionov DA, Gelfand MS: **Identification of a bacterial regulatory system for ribonucleotide reductases by phylogenetic profiling.** *Trends Genet* 2005, **21:**385-389.
49. Torrents E, Grinberg I, Gorovitz-Harris B, Lundstrom H, Borovok I, Aharonowitz Y, Sjoberg BM, Cohen G: **NrdR controls differential expression of the Escherichia coli ribonucleotide reductase genes.** *J Bacteriol* 2007, **189:**5012-5021.
50. Rodionov DA, De Ingeniis J, Mancini C, Cimadamore F, Zhang H, Osterman AL, Raffaelli N: **Transcriptional regulation of NAD metabolism in bacteria: NrtR family of Nudix-related regulators.** *Nucleic Acids Res* 2008, **36:**2047-2059.
51. Partridge JD, Bodenmiller DM, Humphrys MS, Spiro S: **NsrR targets in the Escherichia coli genome: new insights into DNA sequence requirements for binding and a role for NsrR in the regulation of motility.** *Mol Microbiol* 2009, **73:**680-694.
52. Hervas AB, Canosa I, Little R, Dixon R, Santero E: **NtrC-dependent regulatory network for nitrogen assimilation in Pseudomonas putida.** *J Bacteriol* 2009, **191:**6123-6135.

53. Ogasawara H, Ishida Y, Yamada K, Yamamoto K, Ishihama A: **PdhR (pyruvate dehydrogenase complex regulator) controls the respiratory electron transport system in Escherichia coli.** *J Bacteriol* 2007, **189:**5534-5541.
54. Kang Y, Lunin VV, Skarina T, Savchenko A, Schurr MJ, Hoang TT: **The long-chain fatty acid sensor, PsrA, modulates the expression of rpoS and the type III secretion exsCEBA operon in Pseudomonas aeruginosa.** *Mol Microbiol* 2009, **73:**120-136.
55. Kang Y, Nguyen DT, Son MS, Hoang TT: **The Pseudomonas aeruginosa PsrA responds to long-chain fatty acid signals to regulate the fadBA5 beta-oxidation operon.** *Microbiology* 2008, **154:**1584-1598.
56. Mauzy CA, Hermodson MA: **Structural and functional analyses of the repressor, RbsR, of the ribose operon of Escherichia coli.** *Protein Sci* 1992, **1:**831-842.
57. Monterrubio R, Baldoma L, Obradors N, Aguilar J, Badia J: **A common regulator for the operons encoding the enzymes involved in D-galactarate, D-glucarate, and D-glycerate utilization in Escherichia coli.** *J Bacteriol* 2000, **182:**2672-2674.
58. Lee PE, Demple B, Barton JK: **DNA-mediated redox signaling for transcriptional activation of SoxR.** *Proc Natl Acad Sci U S A* 2009, **106:**13164-13168.
59. Park W, Pena-Llopis S, Lee Y, Demple B: **Regulation of superoxide stress in Pseudomonas putida KT2440 is different from the SoxR paradigm in Escherichia coli.** *Biochem Biophys Res Commun* 2006, **341:**51-56.
60. Bordi C, Ansaldi M, Gon S, Jourlin-Castelli C, Iobbi-Nivol C, Mejean V: **Genes regulated by TorR, the trimethylamine oxide response regulator of Shewanella oneidensis.** *J Bacteriol* 2004, **186:**4502-4509.
61. Ansaldi M, Simon G, Lepelletier M, Mejean V: **The TorR high-affinity binding site plays a key role in both torR autoregulation and torCAD operon expression in Escherichia coli.** *J Bacteriol* 2000, **182:**961-966.
62. Yang J, Gunasekera A, Lavoie TA, Jin L, Lewis DE, Carey J: **In vivo and in vitro studies of TrpR-DNA interactions.** *J Mol Biol* 1996, **258:**37-52.
63. Herrera MC, Duque E, Rodriguez-Herva JJ, Fernandez-Escamilla AM, Ramos JL: **Identification and characterization of the PhhR regulon in Pseudomonas putida.** *Environ Microbiol*, **12:**1427-1438.
64. Palmer GC, Palmer KL, Jorth PA, Whiteley M: **Characterization of the Pseudomonas aeruginosa transcriptional response to phenylalanine and tyrosine.** *J Bacteriol*, **192:**2722-2728.
65. Pittard J, Camakaris H, Yang J: **The TyrR regulon.** *Mol Microbiol* 2005, **55:**16-26.
66. Outten CE, Outten FW, O'Halloran TV: **DNA distortion mechanism for transcriptional activation by ZntR, a Zn(II)-responsive MerR homologue in Escherichia coli.** *J Biol Chem* 1999, **274:**37517-37524.
67. Crooks GE, Hon G, Chandonia JM, Brenner SE: **WebLogo: a sequence logo generator.** *Genome Res* 2004, **14:**1188-1190.
68. Gama-Castro S, Jimenez-Jacinto V, Peralta-Gil M, Santos-Zavaleta A, Penaloza-Spinola MI, Contreras-Moreira B, Segura-Salazar J, Muniz-Rascado L, Martinez-Flores I, Salgado H, et al: **RegulonDB (version 6.0): gene regulation model of Escherichia coli K-12 beyond transcription, active (experimental) annotated promoters and Textpresso navigation.** *Nucleic Acids Res* 2008, **36:**D120-124.
69. Novichkov PS, Laikova ON, Novichkova ES, Gelfand MS, Arkin AP, Dubchak I, Rodionov DA: **RegPrecise: a database of curated genomic inferences of transcriptional regulatory interactions in prokaryotes.** *Nucleic Acids Res* 2010, **38:**D111-118.
