# Supplementary material for: Comparative genomic reconstruction of transcriptional networks controlling central metabolism in the Shewanella genus
Source: BMC Genomics. 2011 Jun 15;12(Suppl 1):S3. doi: 10.1186/1471-2164-12-S1-S3 (PMC3223726; doi:10.1186/1471-2164-12-S1-S3)
Supplement: Additional file 6 — Rodionov_AF6.pdf - Experimental validation of NagR regulon in S. oneidensis MR-1. (A) Electrophoretic mobility shift assays to assess NagR protein binding to their predicted DNA operators; (B) Differential regulation of NagR controlled genes determined by quantitative qPCR. [file 1471-2164-12-S1-S3-S6.pdf]

Additional file 6. Experimental validation of N-acetylglucosamine-responsive regulon NagR in *S. oneidensis* MR-1.

(A) Electrophoretic mobility shift assays (EMSA) to assess the interactions of NagR protein (SO3516) from *S. oneidensis* MR-1 with their cognate DNA operators.

(i) Target 49-bp biotin-labeled DNA fragment (0.1 nM) from the *SO3507 (nagK)* upstream region was incubated for 30 min at 37 C with increasing concentrations of NagR (0-100 nM) in the presence or absence of the effector, 20 mM Nag, and analyzed by EMSA. DNA fragment from the upstream region of *SO2489* (*zwf*) gene that lacks a NagR-binding site was used a negative control.

(ii) Target biotin-labeled DNA fragments (1 nM) from the upstream regions of *SO1072* (*cbp*), *SO3507 (nagK), SO3510 (mcp*^{Nag}*),* and SO3514 (*omp*^{Nag}) genes were incubated for 30 min at 37°C with or without 100 nM of the NagR protein, and by EMSA.

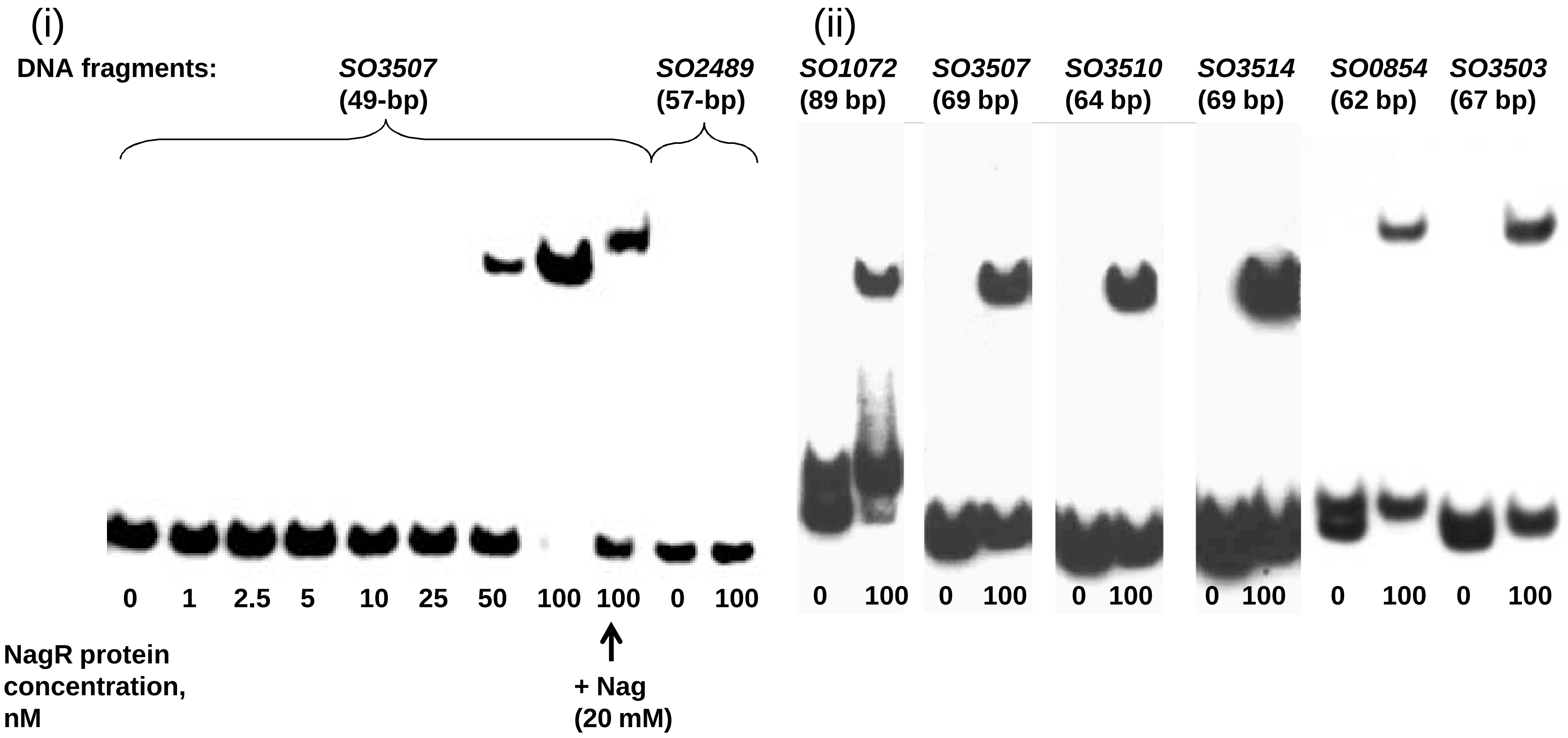

(B) Differential regulation of NagR target genes in *S. oneidensis* MR-1 determined by quantitative qPCR.

Relative transcript levels of the *nagP* (SO3503), *nagK* (*SO3507*), *mcp*^{Nag} (*SO3510*), *omp*^{Nag} (*SO3514*), *cbp* (*SO1072*), *SO0854*, *and zwf* (*SO2489,* used as a negative control*)* genes were measured in *S. oneidensis* MR-1 wild-type and its Δ*nagR* derivative strains grown in the minimal medium supplied with lactate.

Results were normalized to the amount of 16S mRNA.

Fold change was calculated by the $2^{-\Delta CT}$method as a ratio of normalized mRNA levels in Δ*nagR* mutant and wild-type MR-1 strains.

The resulting values are average of two measurements, the standard deviations (S.D.) are given in the last raw.

Tagret gene	SO3503, *nagP*	SO3507, *nagK*	SO3510, *mcp*^{Nag}	SO3514, *omp*^{Nag}	SO1072, *cbp*	SO4635, *mcp*^{Nag} -2	SO0854	SO2489, *zwf*
**Fold change**	**14.5**	**50.2**	**15.8**	**10.6**	**5.3**	1.4	**2.2**	**1.3**
**S.D.**	**1.5**	27	7.1	6.2	2.2	0.2	1.6	**0.1**
